# Common challenges faced in EU-funded projects on integrated care for vulnerable persons

**DOI:** 10.5334/ijic.3104

**Published:** 2017-06-28

**Authors:** Maureen Rutten-van Mölken

**Affiliations:** 1 BMG, NL

## With the rapid increase in the prevalence of multi-morbidity, the need for person-centred integrated care as opposed to fragmented and single-disease focused care has been well recognised

Five EU-funded research initiatives on integrated care for vulnerable people exchanged information and ideas during a workshop at the International Conference on Integrated Care (ICIC) that took place on May 8^th^-11^th^ in Dublin. During this workshop, the project leaders from the initiatives (hereafter called projects) discussed the methodologies and approaches they apply in evaluating, improving, scaling-up and sharing knowledge on best practices, the challenges they face and the potential solutions they consider. The following projects were represented:

1. *Sustainable Tailored Integrated Care for Older People in Europe* (SUSTAIN: http://www.sustain-eu.org/). In SUSTAIN action research is applied to improve established integrated care programmes for older people living at home with multiple health and social care needs. These initiatives include prevention-oriented primary care, home nursing, palliative care, transfer care after hospital discharge and rehabilitative care.
2. *Sustainable Integrated Care Models for Multi-Morbidity: Delivery, Financing and Performance* (SELFIE: http://www.selfie2020.eu/). One of SELFIE’s aims is to strengthen the evidence base of integrated care programmes for persons with multi-morbidity using Multi-Criteria Decision Analysis (MCDA). SELFIE includes promising programmes targeting frail elderly, palliative care- and oncology programmes, programmes for persons with problems in multiple life domains (e.g. medical, housing, financial), and health management programmes that target the entire population in a region.
3. *Advancing Care Coordination and Telehealth deployment at Scale* (ACT@Scale: https://www.act-at-scale.eu/). ACT@Scale uses the Plan-Do-Study-Act cycle to scale-up the implementation of integrated care initiatives deploying tele-health. Among others, it includes programmes on tele-monitoring of diabetes, chronic obstructive pulmonary disease, cardiovascular disease, and psychiatric disorders and on the use of ICT to improve care coordination and ICT to support independent living of frail elderly.
4. *Scaling Integrated Care in Context* (SCIROCCO: http://www.scirocco-project.eu/). SCIROCCO is testing and validating a model, which was developed by the B3 Action Group on Integrated Care of the European Innovation Partnership on Active and Healthy Ageing, to assess the maturity of regions in supporting integrated care. This model scores the maturity of a number of contextual factors on a five-point scale and displays them in a spider web, using an online tool. This model will be tested in a number of real-life scaling-up programmes.
5. *Joint Action on Chronic Diseases* (JA-CHRODIS: http://chrodis.eu/). JA-CHRODIS is embarking on its continuation phase, CHRODIS-PLUS, this autumn. During this phase the project focuses on the implementation of best practices in health promotion, integrated care for diabetes and the multi-morbidity model that were identified and developed in JA-CHRODIS. On the CHRODIS knowledge-sharing platform readers can find excellent chronic disease management practices and policies across Europe, selected on the basis of criteria agreed upon by experts across the EU and an online tool to allow users to evaluate practices, interventions and policies using these criteria.

Although vulnerability is defined differently across the projects, all projects pertain to persons that, in comparison to persons with a single disease, suffer more from: (i) the fragmentation resulting from services being provided by multiple professionals working in different sectors, (ii) the unforeseen effects of treatment interactions, (iii) the lack of flexibility in the application of single-disease guidelines, (iv) the many (conflicting) treatment goals, and (v) the lack of person-centred priority setting based on shared decision making.

## Challenges that the projects face and potential solutions

All five projects focus on promising integrated care programmes for these vulnerable people. These programmes are complex and consist of a mixed package of interacting patient-, provider-, organisational- and financial-oriented interventions that are tailored to the context in which they are introduced and continuously improved as more experience is gained [1]. They have a variety of intended outcomes at different levels and their effectiveness is impacted by the behaviour of those delivering and receiving the interventions and by many contextual factors. As a consequence of this complexity, the EU-funded projects have a number of challenges in common.

One challenge has been *building a common language and a common understanding of methodology* within their consortium. One way to achieve this is by developing conceptual frameworks and common models. Examples include the multi-morbidity model of JA-CHRODIS and the conceptual framework for integrated care in multi-morbidity of SELFIE which can be used to systematically describe, design or improve integrated care programmes. The CHRODIS model focuses more on the care-delivery process itself, whereas the SELFIE framework additionally applies a wider system-level approach. The JA-CHRODIS model is composed of 16 components, grouped in five sections: delivery of the care model system, decision support, self-management support, information systems and technology, and social and community resources. In the SELFIE model the holistic understanding of the person with multi-morbidity in his or her environment is placed in the core. Surrounding this are the micro, meso, and macro layers of six components, i.e. service delivery, leadership and governance, workforce, financing, technologies and medical products, and information and research. Another example is the maturity model of SCIROCCO which has 12 domains, i.e. readiness to change, structure and governance, information & e-health services, finance and funding, standardization & simplification, removal of inhibitors, population approach, citizen empowerment, evaluation methods, breadth of ambition, innovation management and capacity building. More about the models can be found on the websites of the projects. Using such models will contribute to a greater harmonisation of the descriptions, evaluations and implementation strategies across programmes, enabling a better comparison of the specifics of each programme across EU countries.

Another challenge in all five projects has been the *evaluation of the effects of the integrated care programmes* or the effects of the improvement-, scaling-up- and implementation-strategies. In projects like SELFIE where the aim is to contribute to the evidence-base of integrated care programmes, creating a sound research design is a major challenge. Many payers and decision makers still see the randomized controlled trial as the gold standard, but because integrated care programmes involve organizational-, financial-, and health-systems reforms, contamination makes randomization at patient-level impossible. Even randomisation of practices, organisations or regions is often impossible, for example because of historical or policy developments. Hence, they need to be convinced that quasi-experimental designs (in which allocation to intervention and control group is not random) in combination with appropriate statistical techniques to correct for observed confounding (e.g. a combination of propensity score matching/weighting and regression adjustment) and unobserved confounding (e.g. difference-in-difference, instrumental variables, and regression discontinuity methods) still allows causal inference. The Health Economics Special Interest Group (HE-SIG) of the International Foundation for Integrated Care (IFIC) also calls for a wider application of these techniques [5].

In SUSTAIN and ACT@Scale where a participatory approach was used in which the projects actively build working relationships with the integrated care programmes to identify areas for improvement and critical factors for scaling-up, design improvement and scaling-up strategies, and implement these strategies, there is less need to have a control group. The challenge there is to adopt an evaluation approach that monitors the level of adoption of the tailored improvement or scaling-up strategies, and the effects thereof, during the course of the implementation. In SUSTAIN the Evidence Integration Triangle [3] is used and in ACT@Scale the Plan-Do-Study-Act cycle [2], which are both iterative approaches that include regular feedback on progress during the course of the implementation. Similarly, in SCIROCCO, where the usefulness of the maturity model in the transferability of best practices is investigated, and in JA-CHRODIS, where the multi-morbidity model is validated, a participatory approach is adopted.

Although the purpose of measuring outcomes differs between the projects (e.g. effectiveness, readiness to improve, maturity) the EU-funded projects should be applauded for *including patient-reported outcome measures (PROMS), patient-reported experience measures (PREMS)* and in some projects even staff- and manager-reported experience measures. These measures are included in addition to the more commonly used routinely-registered structural and process measures, which are especially relevant to monitor the implementation and upscaling of a programme on an organisational or system level. SELFIE, SUSTAIN, and ACT@Scale have defined a core set or minimum data set of outcomes being measured in all integrated care programmes. This core set is complemented by disease-, cluster-, or programme-specific outcomes. In SELFIE and ACT@Scale the core set is covering the Triple Aim of improving health and wellbeing, improving patients experience with care and reducing costs. In SUSTAIN the core set of outcomes covers the four domains on which SUSTAIN concentrates, which are patient-centeredness, prevention-orientation, safety and efficiency. In ACT@Scale the measurements are divided according to Donabedian’s framework of structure, process and outcome measures, where SCIROCCO’s maturity model is used as indicator of structure. An area of concern that especially applies to projects that evaluate the effectiveness of integrated care programmes is that the time horizon may not be long enough to measure the impact of the initiatives. It depends on the type of programme, but some may need a minimum of five years to prove themselves.

In SELFIE, a broad evaluation framework, called MCDA is applied [4]. This implies that not only the outcomes themselves are measured but also their relative importance. To this end, importance-weights are derived for outcome-concepts and not for specific outcome measures. This will allow these weights to be re-used by others in future MCDAs, using the online MCDA-tool that will be developed. Moreover, the importance is measured from the viewpoint of five different groups of stakeholders, called the 5Ps, referring to Patients, their Partners (or other informal caregivers), Professionals, Payers and Policy Makers. In the MCDA the performance of the programmes on the outcomes is combined with the importance of the outcomes, thus making the relative contribution of the outcomes to the decision-making more explicit and transparent, which can improve the credibility, acceptability, and accountability of the decision-making process about reimbursement from different viewpoints [4].

Including a wide range of outcomes creates *the challenge of collecting, extracting and linking data from different sources.* What appear to be common outcomes may turn out to be defined differently across programmes and even more so across countries. As a solution, several projects have clearly defined outcomes in the core set but allow more variation in the definition of the programme-specific outcomes. Considering the data protection and privacy regulations in each country, the EU-funded projects have generally decided not to build a central patient-level database. Data are stored locally and similar within-country analyses are performed conforming to a common analysis plan, after which cross-country analyses are performed centrally on the aggregated results.

*Being explicit about the policy-decisions that need to be informed by these EU-projects is a challenge in itself.* How can we ensure that the projects address the right policy questions? Involving policy makers from the beginning, when the project is still in the design phase, does not guarantee that the policy-makers’ interests and perspectives are adequately incorporated. Policy makers often have multiple, sometimes conflicting goals, without clear priorities, like increasing access to services, improving person-centeredness, increasing cost-effectiveness, and containing costs within the budget-silos for which they are responsible. The exact decisions that need to be taken are not always clear to them.

This lack of clarity is related to the fact that most of the interventions in the integrated care programmes themselves are usually included in the benefit package of a social health insurance or a national health service, but the additional time and efforts associated with integration-activities like improving communication between professionals, better coordination and continuity of care, involving informal caregivers, supporting self-management, implementing performance-based management, are usually not. So the policy decisions that need to be taken usually pertain to the structural reimbursement or initiation, continuation, extension, and/or wider implementation of these coordinating and management activities to ensure their sustainability in the long run. We need to convince the payers that they should invest in person-centred integrated care in order to reduce the need of other, more expensive, services.

## The added value of EU-funding

Best practices in integrated care are always well-tailored to the specific context for which they are designed. Nevertheless, people can learn a lot from the experience of other countries. EU-funding makes sharing of knowledge possible, it stimulates collaboration and opens doors that would otherwise remain closed. Such funding also increases the general understanding of other health and social care systems and the distinction between generic and context-specific barriers to the implementation of integrated care, thus stimulating the transferability of good practices. Hence, the EU investments provide an opportunity to join forces in finding solutions for the challenge of moving away from a single-disease focussed approach towards person-centred integrated health and social care for the EU citizens most in need. This requires that we, as EU-funded researchers, intensify our interaction and ‘integrate’ our research-silos, as we did during the ICIC workshop.

Prof. Dr. Maureen Rutten-van Mölken, PhD

Professor of Economic Evaluation of Innovative Care for Chronic Diseases

Coordinator of SELFIE and chairman of this workshop at ICIC 2017

School of Health Policy and Management, Institute for Medical Technology Assessment

Erasmus University Rotterdam, the Netherlands

Email: m.rutten@bmg.eur.nl
